# Bioaccessibility of Biofortified Sweet Potato Carotenoids in Baby Food: Impact of Manufacturing Process

**DOI:** 10.3389/fnut.2018.00098

**Published:** 2018-10-23

**Authors:** Claudie Dhuique-Mayer, Adrien Servent, Charlotte Messan, Nawel Achir, Manuel Dornier, Yery Mendoza

**Affiliations:** ^1^QualiSud, Univ. Montpellier, CIRAD, Montpellier SupAgro, Univ. Avignon, Univ. Réunion, Montpellier, France; ^2^CIRAD, UMR QualiSud, Montpellier, France; ^3^Nestlé Research Center, Lausanne, Switzerland

**Keywords:** orange sweet potato, pro-vitamin A, *in vitro* digestion, industrial processes, baby food

## Abstract

Orange-fleshed sweet potato (OFSP), a biofortified crop rich in β-carotene, can be used as a component of baby food recipes in order to tackle vitamin A deficiency in children <6 years old. In this work, the impact of formulation (addition of pumpkin, oil, and egg yolk) and industrial heat processing (pasteurization, sterilization) on carotenoid content and bioaccessibility was evaluated in an OFSP-based baby puree. A commercial OFSP baby food product from Brazil and a homemade OFSP puree were used as references. The losses of *all-trans*-β-carotene ranged from 16 to 21% (pasteurization, homemade) to 32% (sterilization). Because of higher particle sizes and despite a higher content in carotenoids, the homemade puree had a lower bioaccessibility (i.e., micellar transfer using *in vitro* digestion: 0.50%) compared with the sterilized and commercial purees (5.3–6.2%). Taking into account bioaccessibility and applying a 50% conversion to retinol, a 115 g baby portion of the sterilized OFSP-puree formulated with 2% oil provided 31.4% of the daily vitamin A requirement (RDA) for children under 6 years. In comparison, 115 g of homemade OFSP-puree provided only 3.5% of the RDA. Addition of pumpkin to OFSP did not improve the percentage of RDA. Interestingly, the incorporation of an emulsifier (egg yolk powder) before cooking could improve the percentage of provision by a factor of 2.7. These results showed that reaching a balance between formulation and processing is determinant to maximize carotenoid bioaccessibility of carotenoids from OFSP-based baby food.

## Introduction

Biofortified β-carotene OFSP can be an efficient way to deal with Vitamin A Deficiency (VAD). In 2006, it was included in the HarvestPlus challenge program in Uganda and Mozambique (1). Consumption of OFSP can increase in circulating β-carotene and vitamin A body stores (2). The prevalence of VAD in some areas of Brazil was reported to be 21.8% among children under five and remains a public health concern (3–5). Biofortified OFSP varieties such as Beauregard have been developed in Brazil to improve health and nutrition of the population at risk. These varieties are generated by conventional breeding to enhance β-carotene content (6).

The biological activity of β-carotene from OFSP is highly dependent on its bioavailability, i.e. the amount of ingested carotenoid that is available for normal physiological functions in the body including ioconversion to Vitamin A (7). Therefore, the first step of the gastro-duodenal digestion of carotenoids is measured *in vitro* by the bioaccessibility, i.e., the proportion of the micronutrient extracted from the food matrix and made accessible to the enterocytes in a form that would be absorbable (8). Not all the accessible carotenoid will be absorbed, depending on other factor such as dose size or vitamin A status of the individual (9).

The first impact of process on β-carotene bioaccessibility is due to the chemical reactivity of β-carotene. Heat treatments affect its concentration and modify the relative abundance of the *trans* and various *cis* isomers, which have different structures and therefore micellarization capacities.

The other factors influencing carotenoid bioaccessibility are the food matrix microstructure and the interaction with other nutrients that play a key role. Several studies in the past few years have shown that carotenoid bioaccessibility is strongly dependent on the matrix characteristics, and the transformations of the raw material during unit operations such as thermal or mechanical processing. For instance, it is well known that lipids or fibers and their transformation during processing have an influence on the extent of carotenoid micellarisation, but the mechanisms are not clearly elucidated. The majority of studies on this subject are performed with carrots and tomatoes (10).

Although OFSP is a staple crop with a high amount of β-carotene, carotenoid bioaccessibility is rather low in boiled OFSP (0.5–9%) (11–15).

However, addition of 2% soybean oil improved 2–3 fold the micellarization of *all-trans* β-carotene (11) while addition of sunflower oil (2.5%) increased the *all-trans* β-carotene bioaccessibility by a factor 20 (12). Furthermore, the type of oil and the cooking conditions can modify the β-carotene bioaccessibility in OFSP puree (13). Bioaccessibility was particularly enhanced when addition of oil and heating was followed by a homogenization step. The authors of these studies deducted that the preparation method must promote cell-wall rupture; structural information on the food matrix contributed to understand the release of carotenoids from OFSP during food processing and digestion (13).

It is assumed that heat treatments can modulate carotenoid bioaccessibility via disruption of plant cell walls leading to the release of these fat-soluble compounds from protein complexes (16). Some authors have quantified the effects of process parameters on carotenoid bioaccessibility in vegetables such as tomatoes or carrots, (17, 18). However, the effect of industrial processes at different thermal treatments temperatures on carotenoid bioaccessibility in OFSP deserves some more investigation.

Although information about the influence of OFSP varieties and the effect of different cooking methods on carotenoid bioaccessibility is available, the thermal treatment considered is limited to traditional home-made cooking. Consequently, there is a deficit of information on the optimization of carotenoid bioaccessibility considering industrial process parameters such as product core-temperature during heat treatments, pasteurization or sterilization, recipe formulation or the effect of grinding coupled with cooking. Moreover, there is no detailed knowledge about the effect of thermal treatments combined with formulation on carotenoid bioaccessibility in a commercial product compared to a homemade puree.

In this context, the impact of heat processing and formulation on carotenoid content and bioaccessibility was investigated in OFSP-based baby puree in order to evaluate the nutritional interest of industrial treatments (pasteurization, sterilization) according to the typical commercial manufacturing processes. Two thermal treatments were reproduced in the laboratory and compared with a commercial OFSP baby food from Brazil and a home-made OFSP puree. The objective of the study was to understand how the process conditions and the specific baby food formulation affected carotenoid stability and bioaccessibility taking into account cell-structure and particle size.

## Materials and methods

### OFSP roots and pumpkin origin for the preparation of puree

Biofortified OFSP (*Ipomoea batata* (L)-Beauregard variety) and Pumpkin (*Cucurbita Moschata* Duchesne) variety Butternut were grown in Brazil. The OFSP and Butternut samples were purchased from a known Nestlé supplier (OFSP) and from the open market (Butternut). The samples were purchased at the beginning of March 2017. Harvested roots and pumpkin from Sao José de Rio Pardo (State of Sao Paulo) at marketable quality were sent by plane to the Cirad laboratory in France. Roots or pumpkin were immediately peeled, cut in equal-sized pieces (1 × 2 × 1 cm) and stored at −20°C under vacuum until analysis.

### Industrial treatments

**Figure 1:**
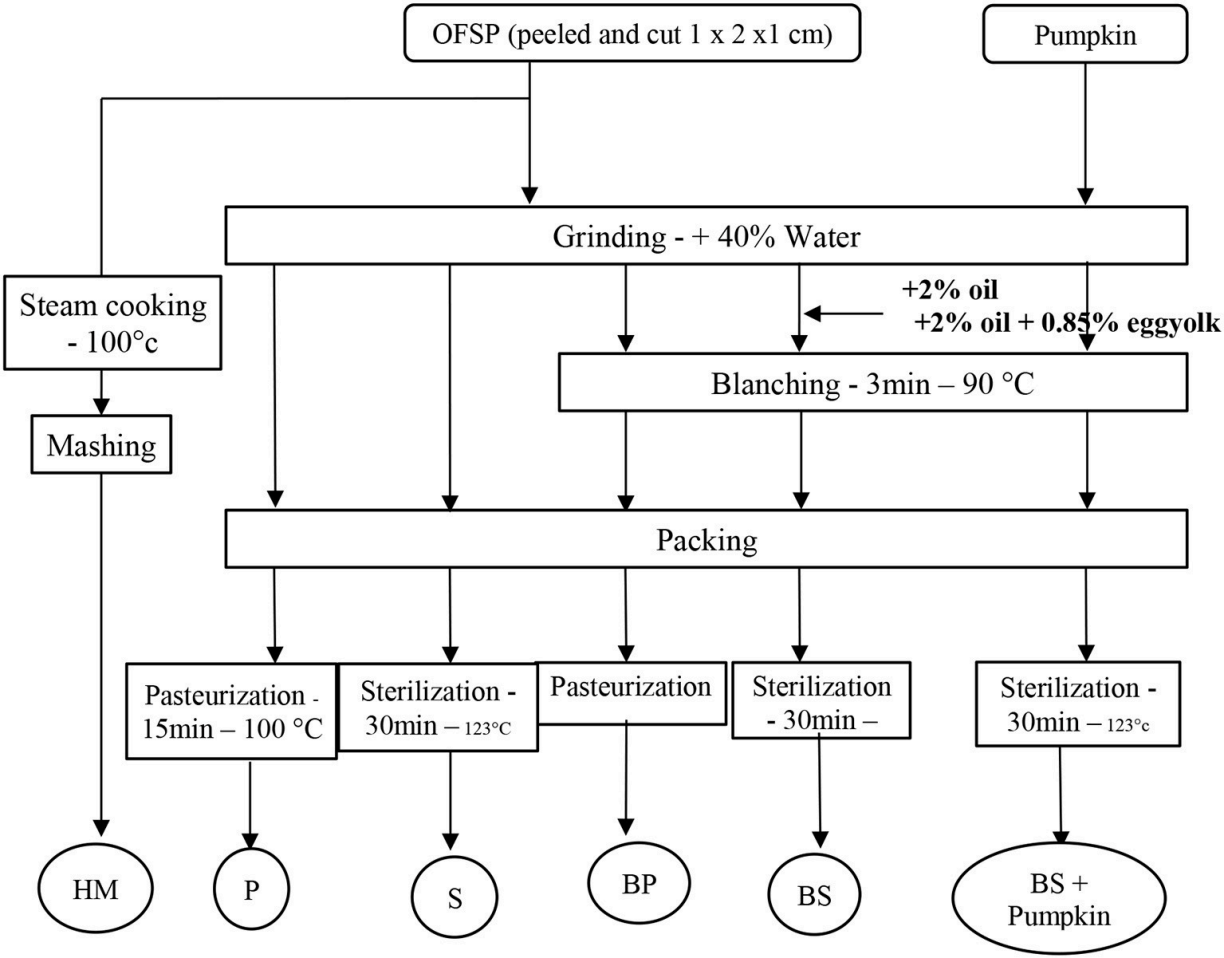
Schematic overview of the experimental process. HM, Home-made Process; BP, Blanching and Pasteurized or BS, Blanching and Sterilized; P, pasteurized, or S, Sterilized.

Small pieces were ground in a heating blender (Thermomix®-Vorwerk, Wuppertal, Germany) with 40% of water according to the industrial baby puree manufacturing process (Nestlé). To make a fine puree, grinding was set for 3 min at maximum power. Following grinding the product was submitted to blanching during 3 min at 90°C. The puree was then potted (baby food pot 106 ml) before heat treatment. A flow chart of the experimental process describing the typical industrial manufacturing processes is shown in Figure 1. Industrial treatments were carried out according to the typical industrial manufacturing processes (Figure 1). Pasteurization was carried out in a thermostatic water-bath during 15 min at 100°C. Sterilization was carried out in an autoclave (Auriol SA, Marmande, France) for 30 min at 123°C according to the factory conditions (Nestlé-Sao Jose do Rio Pardo). For each thermal treatment, an Almemo® probe 2690 (Ahlborn, Holzkirchen, Germany) with a standard K thermocouple was used to record the temperature in the puree during the process. Sterilization and pasteurization values, F_0_ and P_0_, were classically calculated from Equation (1) using 121.1 and 70°C as reference temperatures *Tr* and a *z* factor of 10°C for both. The numerical Euler's method was used as usual for the integration from temperature measurements T at the geometric center of the pot vs. time *t*.

(1) $$F_{0}\;or\;P_{0}=\int_{0}^{t}10^{\frac{T-Tr}{z}}dt$$

### Home-made cooking

OFSP pieces (1 × 2 × 1 cm) were cooked for 7 min in a home-steamer (Coveniant-SEB, Stockholm, Sweden). Small pieces were then crushed coarsely using a home-fine grinder type small vegetable mill (Moulinex, Ecully, France). Temperature was monitored with an Almemo® probe 2690 (Ahlborn, Holzkirchen, Germany) with a standard K thermocouple.

### Puree formulation

Mixed samples: OFSP and pumpkin puree was prepared by adding 50% OFSP puree to 50% pumpkin puree. OFSP puree with oil: according to industrial recipes, 1.2% of rapeseed oil and 0.8% of corn oil were added after 3 min grinding. OFSP puree with oil and emulsifier: 0.85% of e dehydrated egg yolk (powder) was added to the oil for final sample preparation.

### Granulometry and light microscopy

Particle size measurement was determined by LASER diffraction using a Malvern Mastersizer (Mastersizer 3000, Malvern Instruments Limited, Worcestershire, UK). Size distributions (volume or mass fractions against particle size) were calculated. The weight average sizes of the respective proportions (50 or 90% of total volume or mass) were expressed as D_50_ or D_90._ Samples were observed by light-microscopy after Lugol's iodine 5% dyeing in order to reveal starch granules. Starch, i.e., amylose, cause an intense blue-violet coloration in the presence of Lugol's solution. Observations were achieved using a DM 6000B microscope (Leica, Wetzlar, Germany) with transmitted light and a head camera Nikon DS Ri-2 (Nikon, Tokyo, Japan).

### Carotenoid analysis

Carotenoid extraction from OFSP or pumpkin puree was carried out as follows: a sample of puree (0.250 g) was homogenized with 5 mL of distilled water extracted twice with 20 mL of ethanol/hexane (4:3, v/v). β-Apo-8'-carotenal was added as an internal standard. The residue was separated from the liquid phase by filtration with a filter funnel (porosity n°2). Ethanol (30 mL) and hexane (30 mL) were successively used to wash the residue. Organic phases were transferred to a separator funnel. The aqueous layer was removed. The hexane phase was dried using anhydrous sodium sulfate and filtered before evaporation to dryness under vacuum at 37°C. Carotenoid extracts were dissolved in 1 ml of 50/40/10 (v/v/v) mixture of dichloromethane, methyl-*tert*-butyl-ether (MTBE) and methanol, and analyzed by HPLC.

Carotenoid extraction from digested puree was carried out according to Poulaert et al. (15). An aliquot of digested sample (10 ml) was extracted three times with 10 ml of hexane and 5 ml of ethanol spiked with 100 μl of β-apo-8'-carotenal as recovery standard. The pooled hexane extracts were evaporated and re-dissolved in 500 μl of mobile phase (250 μL of dichloromethane and 250 μL of an 80:20 (v/v) mixture of methyl tert-butyl ether (MTBE) and methanol).

HPLC analysis of carotenoids: Carotenoids were analyzed by reverse-phase-HPLC using an Agilent 1100 system (Massy, France), along a C_30_ column (250 × 4.6 mm i.d., 5 μm YMC (Europ GmbH, Dinslaken Germany). The mobile phases were: H_2_O as eluent A, methanol as eluent B and MTBE as eluent C at 1 mL.min^−1^ flow rate. The column temperature was 25°C, and the injection volume was 20 μL. The gradient was: 0–5 min, 40% A, 60% B (initial condition); 5–10 min, 20% A, 80% B; 10–60 min, 4% A, 81% B, 15% C; 60–71 min, 4% A, 11% B, 85% C; 71–72 min 100% B, and back to the initial condition for re-equilibration. Absorbance was monitored using an Agilent 1100 photodiode array detector. Carotenoid quantification was achieved by calibrating β-carotene and lutein at 450 nm (correlation coefficients of 0.994 and 0.998, respectively).

### *In vitro* digestion

The *in vitro* digestion model was described previously by Dhuique-Mayer et al. (19) and was first optimized by Reboul et al. (20). We chose this model because these authors optimized the procedure regarding pH and incubation times by taking data on lipid digestion and carotenoid processing *in vivo* into account. The *in vitro* static model used in this study has been validated against human studies and is considered a reliable model for carotenoid behavior (21).

OFSP Puree samples (15 g) were mixed in 32 mL of saline solution (NaCl 0.9%) and homogenized for 10 min at 37° C in a shaking water bath. To mimic the gastric digestion step, the pH was adjusted to 4.0 with 1 M NaOH, after which 2 mL porcine pepsin Sigma P7012- 2,500 units/mg protein (40 mg.mL^−1^ in 0.1 M HCl) was added. The homogenate was incubated at 37°C in a shaking water bath for 30 min. To mimic the intestinal digestion step, the pH of the gastric mixture was raised to 6.00 ± 0.02 by adding 20 mL of 0.45 M sodium bicarbonate pH 6.0. Subsequently, 9 mL of a mixture containing 2 mg.mL^−1^ pancreatin (Sigma P1750 4 x usp) and 12 mg.mL^−1^ bile extract (Sigma B8631) in 100 mmol.L^−1^ trisodium citrate, pH 6.0, and 4 mL bile extract at 0.1 g.mL^−1^ were added. The samples were incubated in a shaking water bath at 37°C for 30 min to complete the digestion process. Micelles were separated by centrifugation (48,000 g for 4 h at 10°C using a Beckman-Coulter Avanti-JE- JA 21 rotor, Villepinte, France) and the aqueous fraction was collected and filtered through a 0.22 μm filter (Millipore). Aliquots were stored at −20 °C under nitrogen until analysis.

### Statistical analysis

Statistical analyses were performed using the XLSTAT software (version 2016). The variance homogeneity was evaluated using the Bartlett test. Analysis of variance (ANOVA) was used to test differences in mean *in vitro* bioaccessible carotenoids values. Where ANOVA indicated significant differences between the treatments, a Tukey's honestly significant difference multiple rank test was used to further analyze these differences.

## Results and discussion

### Impact of processing on carotenoid content in OFSP puree

**Figure 2:**
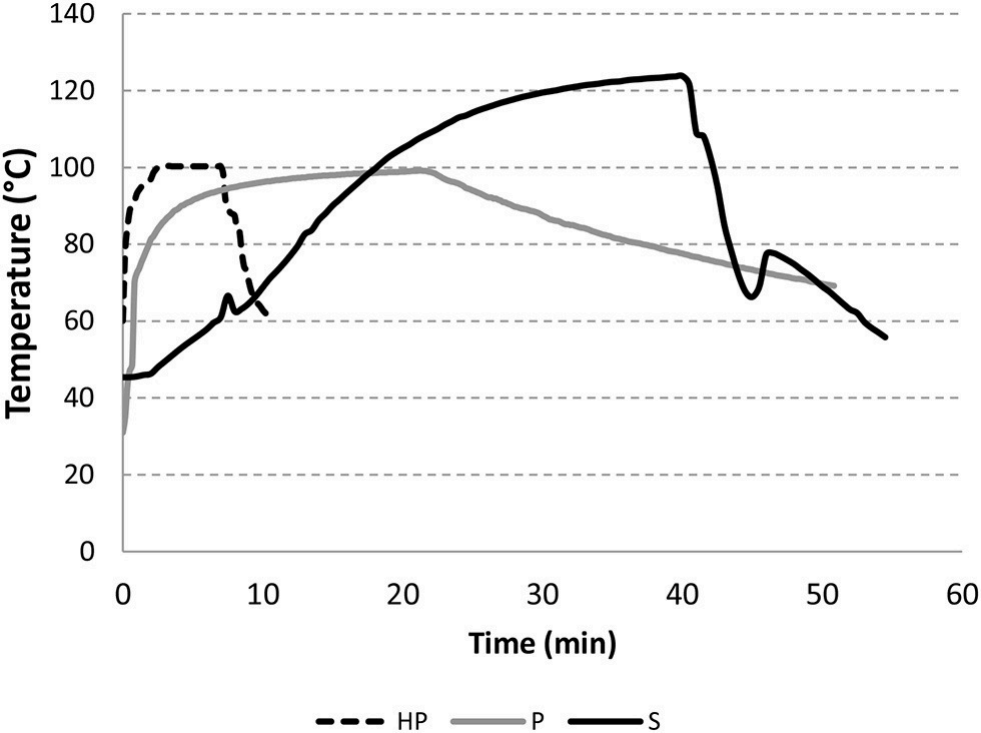
Evolution of temperature during pasteurization (P), sterilization (S) and home-made (HP) processes.

Product temperature was monitored for 7–50 min, as appropriate for each of the 3 experimental processes: homemade, pasteurization and sterilization (Figure 2). In the operating conditions used, pasteurization values P_0_ for industry-like pasteurized (P) purees was 8,628 min. For the sterilized samples, the sterilization value F_0_ was 16.1 min. The high sterilization and pasteurization values applied in the industrial treatments are needed in order to cook the product as required for microbiological stabilization.

The losses of *all-trans*-β-carotene ranged from 16.7% (blanched/pasteurized) to 32.5% (blanched/sterilized). Homemade process and pasteurization led to the same loss of *all-trans*-β-carotene (around 19%) while sterilization led to 32% loss. As expected, carotenoid content and losses in the industrial sample were quite similar to those of the laboratory sterilized puree. No losses of *all-trans*-β-carotene were observed for the step of blanching. So, this weak thermal treatment is clearly insufficient to impact the carotenoid content of the puree. Regarding 13-cis-β*-*carotene, its proportion over total carotenoids increased in sterilized products (4.3–7.2%) especially for blanched sterilized (BS) and industrial samples. The higher content of 13-cis-β-carotene is probably the result of the addition of thermal treatment steps (sterilization) (Table 1).

**Table 1:** Carotenoids contents in OFSP puree and retention after thermal treatments.

***all*****-trans-*****β*****-carotene**				**13-cis-*β-*carotene**		
**Treatments**	**(μg/g FW)**	**(μg/g DW)**	**% losses**	**(μg/g FW)**	**(μg/g DW)**	**% of total β-carotene**
HM	98.5 (6.1)^a^	277 (17)	21.3^b^	3.8 (0.7)^a^	10.8 (1.9)	3.7^d^
P	62.2 (5.2)^b^	284 (23)	19.4^b^	–	–	–
BP	63.1 (0.6)^b^	293 (3)	16.7^b^	1.9 (0.1)^b^	9.2 (0.1)	3.0^e^
S	48.9 (0.4)^c^	243 (2)	31.1^a^	2.2 (0.1)^c^	11.1 (0.3)	4.3^c^
BS	48.8 (0.3)^c^	237 (2)	32.5^a^	2.6 (0.1)^c^	12.6 (0.4)	5.1^b^
Commercial baby food	54.8 (2.3)^c^	260 (10)	^_^	4.3 (0.3)^d^	20.3 (1.3)	7.2^a^

*HM, Home-made Process 0% added water; BP, Blanching and Pasteurized or BS, Blanching and Sterilized; P, pasteurized or S, Sterilized. For all these processes 40% added water. Data are means of four independent determinations (SD)-Values in the same column with the same superscripts were not significantly different at p < 0.05 (comparable within first line or second line)*.

Through these thermal degradation pathways, carotenoid losses could be forecast assuming first order kinetics and Arrhenius behavior. Using kinetic parameters found in Penicaud et al. (22) for pumpkin puree treated between 60 and 100°C (Ea = 27 kJ mol^−1^, ${\text{k}}_{60{}^{\circ}\text{C}}$ = 4. 10^−3^ min^−1^ for home-made and pasteurized purees, ${\text{k}}_{100{}^{\circ}\text{C}}$ = 8. 10^−3^ min^−1^ for the sterilized one), the calculated carotenoid losses were 33% for sterilization, 16% for pasteurization, and 9% for home-made puree. Predictions were very close to the experimental values for sterilization as well as for pasteurization. Nevertheless, calculation underestimated losses during the homemade treatment (experimental losses 21%). This difference could be related to additional degradation pathways that also involve oxidative mechanisms because the homemade puree was cooked in an open system in direct contact with air.

Losses of *all-trans*-β-carotene in boiled OFSP depended on the cooking and preparation method. Failla et al. (11) reported only 11% losses but roots were just cut in two parts prior to thermal treatment, suggesting that boiling in small cubes or mashed puree as in the present study leads to more degradation because of the higher area exposed. However, other authors observed on average 34% of losses with lower cooking time and a similar preparation (13). Recently, others authors reported that boiling OFSP decreased total provitamin A by 15% due in large part to conversion of *all-trans*-β-carotene to 13-*cis*-β*-*carotene (6).

### Carotenoid bioaccessibility changes in OFSP purees submitted to different processes

**Figure 3:**
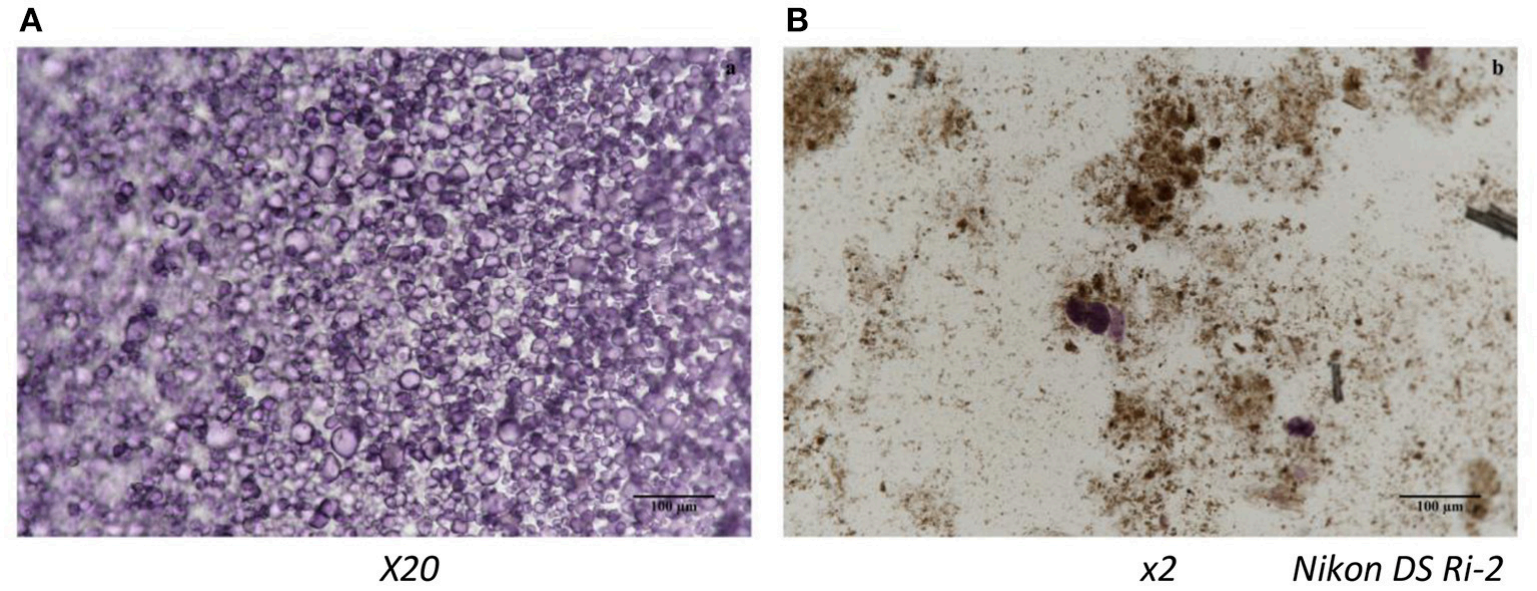
Light Microscopy image showing the microstrucutre of starch granules in OFSP puree (Lugol's test): **(A)** without blanching sterilized and **(B)** blanching sterilized.

Data reported in Table 2 shows the percentage of carotenoids transferred from the OFSP puree into the micelles after *in vitro* digestion (i.e., the bioaccessibility). In general, bioaccessibility of *all-trans*-β-carotene was higher in sterilized purees (4.11–6.34%) compared to pasteurized and home-made puree. The relatively low bioaccessibility of *all-trans*-β-carotene from boiled OFSP was previously observed by Failla et al. (11), from 0.6 to 3%, or by Bengtsson et al. (12), from 0.5 to 1.1% depending on cultivars. The better results obtained in our case could be due to homogeneization, which was made prior to thermal treatments. This was a recommendation made by Bengtsson et al. (13). Moreover, it is clear that blanching followed by sterilization enhanced *all-trans*-β-carotene bioaccessibility. Note that the micellarization efficiency was better for the isomer *13-cis*-β-carotene than for *all-trans*-β-carotene in sterilized purees (59.7–60%). This is in agreement with previous data and is due to the carotenoid structure allowing a better incorporation in micelles (11–14), also in agreement with previous data, irrespective of the food matrix (23). The best thermal treatment was blanching followed by sterilization (6.34% for experimental treatment and 5.32% for the factory sample Nestle). Our results clearly indicate that the step of blanching improved carotenoid bioaccessibility. Microscopic observation of puree microstructure showed intact starch granules in sterilized samples without blanching (Figure 3). According to Brackman et al. (24), a large part of the carotenoids in OFSP is in crystalline form and presents different cluster aggregates surrounding the starch granules. These authors also studied the effect of thermal treatment on carotenoid and starch granule morphology using the CARS/SHG microscopy technique in OFSP and concluded that, despite a change in starch morphology, carotenoids remained intact after boiling induced a release of them into cellular lipid droplets. In our study, the combined treatments of grinding and heating during the blanching step could disrupt totally the starch granules, as can be seen on Figure 3B.

**Table 2:** Carotenoid bioaccessibility in different thermal treatments and formulation of OFSP puree.

	**Carotenoids bioaccessibility (%)**		
	***all*-trans-β-carotene**	**α-carotene**	**13-*cis*-β-carotenearotene**
**TREATMENTS OFSP**			
HM	0.50 (0.03)^a^	-	6.44 (0.63)^a^
P	0.99 (0.10)^a^	-	-
BP	3.65 (0.70)^b^	-	28.99 (4.35)^b^
S	4.11 (0.23)^b^	-	60.02 (3.82)^c^
BS	6.34 (1.27)^c^	-	59.88 (6.30)^c^
Commercial baby food (OFSP)	5.32 (0.12)^c^	-	59.73 (1.52)^c^
**FORMULATION**			
Pumpkin^1^	14.5 (0.1)	19.2 (1.4)^x^	52.3 (4)^d^
BS + pumpkin^1^	4.0 (0.8)^b^	19.0 (0.5)^x^	50.8 (9)^d^
Baby food Nestle (pumpkin^2^)	5.0 (0.9)^c^	6.5 (1.0)^y^	24.5 (5.5)^b^
BS + oil (2%)	25.1 (4.3)^d^		22.4 (5.1)^b^
BS + oil (2%) + emulsifier (Egg yolk powder 0.85%)	43.7 (1.5)^e^		48.8 (0.9)^d^

*HM, Home-made Process; BP, Blanching and Pasteurized or BS, Blanching and Sterilized; P, pasteurized or S, Sterilized. Pumpkin puree was blanching and sterilized with 40% added water. Pumpkin^1^and pumpkin^2^ were different and were blanched-sterilized. Data are means of three independent determinations (SD)-Values in the same column with the same superscripts were not significantly different at p < 0.05 (comparable within first line or second line)*.

**Figure 4:**
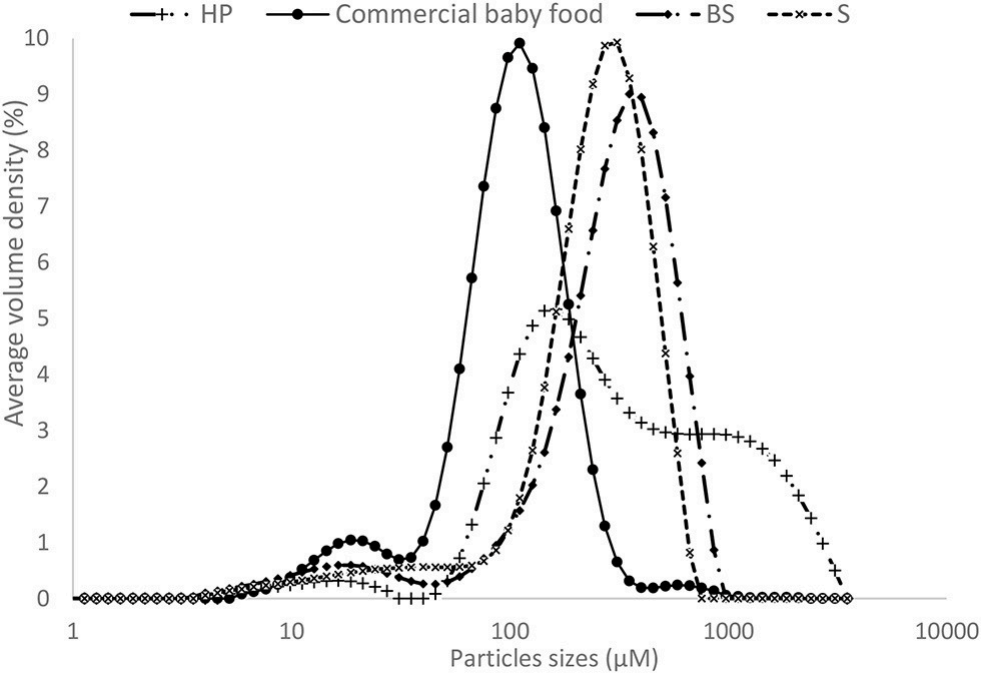
Particle size distribution of the different processed OFSP purees.

Despite a higher content of carotenoids, the homemade puree showed a lower carotenoid micellarization (0.50%) compared with sterilized and commercial purees (5.32–6.34%). Regarding the granulometry, the particle size distribution of the different processed purees (Figure 4), was inversely correlated to carotenoid bioaccessibility, particularly for homemade puree (D_90_ = 1580 μm) compared with other processed purees. However, the lower particle size of the industrial sample (Nestlé factory) (D_90 =_ 2 10 μm) didn't show a different carotenoid bioaccessibility compared to other experimental sterilized purees (D_90_ = 555 μm on average). This implies that for an equivalent thermal treatment such as sterilization, particle size from 555 μm on average was sufficient to obtain the maximum carotenoid bioaccessibility in OFSP puree. Several studies on carrots show that mechanical combined to thermal treatments are important to reduce particle size, which is one of the key factors influencing carotenoid bioaccessibility (17, 25, 26). These authors concluded that to achieve high β-carotene bioaccessibility, intense thermal processing such as sterilization is required (whatever the mechanical disruption) or mild thermal processing must be combined with intense mechanical processing (25).

### Carotenoid bioaccessibility changes in OFSP purees submitted to different formulations

In order to know the impact of formulation, other vegetables or ingredients were added to OFSP puree according to the typical industrial manufacturing recipes used in the Brazilian factory. For that, we chose pumpkin, another biofortified vegetable rich in carotenoids grown in Brazil. Some ingredients such as rapeseed oil and corn oil (2%) are necessary to reach a nutritional balance for baby food. The addition of a natural emulsifier like egg yolk powder (0.85%) allows stabilization and homogenization of the final product. β-carotene bioaccessibility of pumpkin was 2.3-fold higher than that of OFSP with the same thermal treatment (blanching and sterilization). However, when pumpkin and OFSP were mixed 50/50 in a puree (blanched and sterilized) β-carotene micellarization strongly decreased to 4%. It was clear that incorporation of OFSP did not improve β-carotene bioaccessibility. Conversely, addition of pumpkin to the recipe did not give a greater efficiency of micellarization for β-carotene, suggesting that the mixture of matrices causes a problem concerning bioaccessibility. The bioaccessibility difference between β-carotene from butternut and from OFSP could be explained by carotenoid substructures localized in plant chromoplasts. Jeffery et al. (27) compared cell wall and chromoplast substructures in butternut and sweet potato. Sweet potato carotenoids in chromoplast substructures were in crystalline form, with few plastoglobules or with disorganized membrane bound structures giving poor bioaccessibility, while butternut squash chromoplast substructures presented plastoglobules, tubules and vesicle forms leading to a better carotenoid release and thus better bioaccessibility. However, compared to others fruits such as mango or papaya, which contained a highest proportion of carotenoid in plastoglobule form, bioaccessibility of these two vegetables were relatively low; the explanation offered is that tubules and vesicles, which contain protein-bound carotenoids, act as a barrier for carotenoid release during digestion (28). That being said, our results on β-carotene bioaccessibility from butternut squash were higher than that those seen by other authors which applied heat treatment prior to grinding and homogenization and found β-carotene bioaccessibility <4% (29, 30). As previously said for OFSP, the higher percentage of carotenoid bioaccessibility from butternut obtained in our study (14–19%) could be because the samples were subject to grinding was made before the thermal treatments.

As expected, the presence of oil (2%) during the thermal treatment of OFSP puree resulted in an increase of the incorporation of β-carotene into micelles from 6.3 to 25.1%. Interestingly, the β-carotene transferred into the micellar fraction increased to 43.7% when egg yolk was added. It is now well known that addition of fat improves carotenoid bioaccessibility and bioavailability (10). The addition of oil during heating had a positive effect on carotenoid micellarization as underlined previously by Bengtsson et al. (13). The addition of 2.5% oil after processing and before *in vitro* digestion did not improve carotenoid bioaccessibility in the study carried out by Berni et al. (6). These authors showed a low efficiency of micellarization of all-*trans* β-carotene with addition of oil from 4 to 8% compared to that of our study (25.1%), whatever the cultivar or the type of heat treatment used in the cooking. Finally, a little amount of the egg yolk powder combined with 2% oil increased again the percentage of carotenoid bioaccessibility from 25.1 to 43.7%. Egg yolk powder is a natural emulsifier containing lipids: 66% triacylglycerides, 28% phospholipids (mainly phosphatidylcholine) and 6% cholesterol (31). Solely based on lipid addition, it is understandable that carotenoid bioaccessibility increases. However, phosphatidylcholine has a key role in the carotenoid bioaccessibility increase by acting as a surfactant to improve micelle formation, enhancing the incorporation of β-carotene. Verrijssen et al. (32) showed that β-carotene bioaccessibility increased with increasing phosphatidylcholine concentration in carrot-based model emulsion.

### Effect on each step and combination of process/formulation

From experimental concentrations and bioaccessibilities obtained in the different processing/formulation combinations tested, we were able to evaluate the individual impact of each step. This impact was quantified through the multiplying coefficient that compared the product after and before each step X_after_/X_before_, with X the carotenoid concentration or bioaccessibility or a combination of both. Our calculations are based on the assumption that the coefficient measured after several steps is the product of the coefficients due to each step alone (Table 3).

**Table 3:** Impact of each processing and formulation step on concentration (C) and bioaccessibility (B) of the two main carotenoids of OFSP puree (home-made puree used as initial reference).

**Multiplying coefficient (X_after_/X_before_)**	***all*****- trans** β**-carotene**			**13-*****cis-***β**-carotene**			**Vitamin A equivalent**
	**C**	**B**	**CxB**	**C**	**B**	**CxB**	
**PROCESSING**							
Blanching	1.00	2.62	2.62	1.17	1.00	1.17	1.45
Pasteurization	0.63	1.98	1.25	–	–	–	–
Sterilization	0.50	8.22	4.11	0.58	9.32	5.41	4.34
**FORMULATION**							
Addition 2% oil	0.38	3.96	1.50	2.35	0.37	0.87	1.4
Addition egg yolk	1.03	1.74	1.79	1.55	2.18	3.38	2.97

From a processing point of view, these coefficients confirm that sterilization is the most important step to improve nutritional quality through carotenoid bioaccessibility. Despite the fact that thermal damage at sterilization temperatures degrades almost half of the original carotenoid content, these losses are largely compensated by the almost tenfold increase in bioaccessibility. The two major carotenoids, β-carotene and its 13-cis-isomer, showed the same behavior and concentrations multiplied by bioaccessibilities finally increased by 4–5. To a lesser extent, the same trends were noticed for pasteurization, although the bioaccessibility increase was not sufficient to offset thermal damages. Another heat treatment, blanching—which requires a much lower temperature—was also interesting because it doubled the bioaccessibility of β-carotene without affecting its concentration. Regarding the recipe formulation, addition of oil decreased *all-trans*-β-carotene and increased 13-*cis*-β-carotene content. These results were in accordance with literature reports noticing a higher cis-isomerization in non-polar solvent or oils (33). Nevertheless, because it helped micellarization, losses in β-carotene were compensated by the bioaccessibility rise. Surprisingly, bioaccessibility of 13-*cis-*β-carotene was reduced after addition of oil probably because the high content of isomers was difficult to micellarize totally, which leads to a saturation phenomenon. As already explained, addition of egg yolk doubled carotenoid bioaccessibility thanks to its emulsifying property and its lipophilic nature. Finally, as expected, the interest to combine heat processing and formulation was clearly highlighted in this work. The best results in terms of vitamin A equivalents were obtained by coupling blanching and sterilization with addition of oil and egg yolk. This combination multiplied the global nutritional quality by 26. The combination blanching/sterilization with addition of egg yolk but no oil was also interesting (multiplied by 19) compared to the addition of oil only (multiplied by 9).

### Contribution of OFSP purees to the daily vitamin a requirement (rda) for children under 6 Years

**Figure 5:**
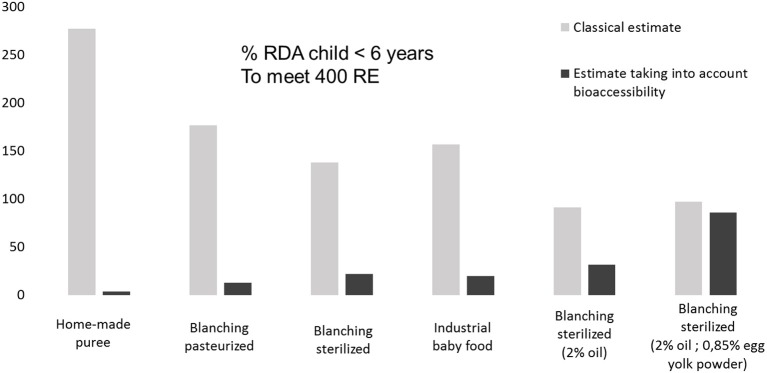
Differences between estimates of vitamin A (% RDA child <6 years) in 115 g portion OFSP processed purees using classical estimate RAE from food (Retinol Activity Equivalent-μg *trans*-β-carotene/12 and 13-*cis*-β-carotene/24) and estimate taking into account bioaccessibility (calculated with RE: Retinol Equivalent μg *trans*-β-carotene/6 and 13-*cis*-β-carotene/12).

Estimations of the vitamin A activity expressed in percentage of RDA in OFSP-based baby food purees were calculated using a classical estimate from food or by taking into account bioaccessibility. The results reported in Figure 5 showed that classical estimates of vitamin A activity from food are usually overestimated compared with calculations taking into account bioaccessibility. Taking into account bioaccessibility and applying a 50%, conversion to retinol (34) shows that a 115 g baby portion of the OFSP-puree with 2% of added oil or emulsifier could provide 31.4 or 85.5% of the daily vitamin A requirement (RDA) for children under 6 years. In comparison, 115 g of OFSP-puree homemade would provide only 3.5% of the RDA. The industrial sample and the laboratory sterilized puree provided similar percentages of RDA (19.7 and 21.8%, respectively). Note that the commercial sample did not contain any fat. Our results support the formulation proposed by the industry, i.e., to add 2% of oil and 0.85% of egg yolk powder. This work shows that the commercial process and formulation of OFSP baby food puree provides more bioavailable carotenoids than homemade puree. In other words, adequate processing can largely improve bioavailability. These results corroborate the findings of Edwards et al. (35), who studied carrot baby food and demonstrated that a commercial puree contained more bioavailable carotenoids than boiled mashed carrots (derived from the same lot of carrots).

## Conclusion

Our study shows that the industrial process to manufacture baby food has a highly positive impact on OFSP carotenoid bioaccessibility, much higher than that of the homemade preparation. The combined effect of each process step and the formulation provides better results in terms of vitamin A equivalents and thus a higher nutritional quality. Moreover, our results support the formulation of OFSP proposed in the industrial samples we tested, not only to improve the overall safety and quality of the baby-food product but also, in particular, the carotenoid bioaccessibility. Based on this work, we recommend the development of functional baby food from OFSP puree as a way to reduce the risk of vitamin A deficiency and improve public health.

## Author contributions

CD-M designed the research and was responsible of nutrition tasks. AS and CM performed the experimental work. MD and NA were responsible of the processing design. YM was the project initiator and corrected the english language.

### Conflict of interest statement

YM is employed by the Nestlé Company (Switzerland). The remaining authors declare that the research was conducted in the absence of any commercial or financial relationships that could be construed as a potential conflict of interest.
